# Force-Induced Unfolding Simulations of the Human Notch1 Negative Regulatory Region: Possible Roles of the Heterodimerization Domain in Mechanosensing

**DOI:** 10.1371/journal.pone.0022837

**Published:** 2011-07-28

**Authors:** Jianhan Chen, Anna Zolkiewska

**Affiliations:** Department of Biochemistry, Kansas State University, Manhattan, Kansas, United States of America; Massachusetts Institute of Technology, United States of America

## Abstract

Notch receptors are core components of the Notch signaling pathway and play a central role in cell fate decisions during development as well as tissue homeostasis. Upon ligand binding, Notch is sequentially cleaved at the S2 site by an ADAM protease and at the S3 site by the γ-secretase complex. Recent X-ray structures of the negative regulatory region (NRR) of the Notch receptor reveal an auto-inhibited fold where three protective Lin12/Notch repeats (LNR) of the NRR shield the S2 cleavage site housed in the heterodimerization (HD) domain. One of the models explaining how ligand binding drives the NRR conformation from a protease-resistant state to a protease-sensitive one invokes a mechanical force exerted on the NRR upon ligand endocytosis. Here, we combined physics-based atomistic simulations and topology-based coarse-grained modeling to investigate the intrinsic and force-induced folding and unfolding mechanisms of the human Notch1 NRR. The simulations support that external force applied to the termini of the NRR disengages the LNR modules from the heterodimerization (HD) domain in a well-defined, largely sequential manner. Importantly, the mechanical force can further drive local unfolding of the HD domain in a functionally relevant fashion that would provide full proteolytic access to the S2 site prior to heterodimer disassociation. We further analyzed local structural features, intrinsic folding free energy surfaces, and correlated motions of the HD domain. The results are consistent with a model in which the HD domain possesses inherent mechanosensing characteristics that could be utilized during Notch activation. This potential role of the HD domain in ligand-dependent Notch activation may have implications for understanding normal and aberrant Notch signaling.

## Introduction

**Figure 1:**
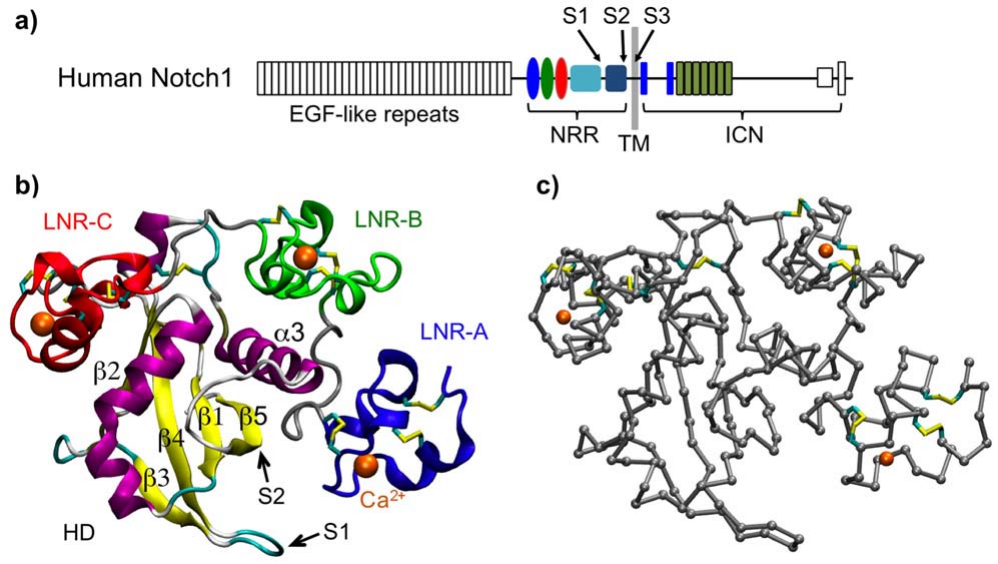
Overview of the human Notch1 domain organization and structure. a) The domain organization of human Notch1 receptor. The N-terminus includes 36 EGF-like repeats, followed by the NRR, a short transmembrane (TM) segment, and the ICN. Approximate locations of the proteolytic cleavage sites S1, S2 and S3 are marked. b) The X-ray structure of human Notch1 NRR (PDB:3eto [22]). The N-terminal LNR domains A–C are colored in blue, green and red, respectively. All disulfide linkages and three bound calcium ions are shown. The HD domain is colored based on the local secondary structure, with the S2 cleavage site and adjacent secondary structural elements (β1, β5 and α3) marked. c) A Cα-only model of human Notch1 NRR derived from the X-ray structure.

Notch signaling is a highly conserved inter-cellular communication pathway that plays critical roles in embryonic development and in tissue homeostasis [1]–[3]. Abnormal Notch signaling is frequently associated with a number of human diseases, including T-cell acute lymphocytic leukemia (T-ALL) and solid tumors [4], [5]. At the heart of Notch signaling are the Notch receptors, a family of highly modular, single-pass transmembrane proteins [6], [7]. There are four Notch receptors in mammals, designated as Notch1–4. All Notch receptors have a similar modular architecture, as illustrated in Fig. 1a for human Notch1. During maturation, the Notch receptor is cleaved at the S1 site by a furin-like protease, which yields a heterodimer with non-covalently associated extracellular and transmembrane subunits [8], [9]. Canonical Notch activation is initiated by binding of a transmembrane ligand of the Delta/Serrate/LAG-2 (DSL) family on the signal-sending cell to a Notch receptor on the surface of the signal receiving cell [2], [6], [7]. Through mechanisms yet to be precisely determined, ligand engagement triggers regulated intra-membrane proteolysis where the Notch receptor is first cleaved at a juxtamembrane extracellular site S2 by ADAM10 or ADAM17 metalloproteases [10]–[14]. After the S2 cleavage, the Notch receptor is further cleaved at an intra-membrane S3 site by the γ-secretase complex [15]–[17]. The S3 cleavage releases the intracellular portion of Notch (ICN) and allows it to translocate to the nucleus and participate in transcription regulation in a context and gene dose dependent manner [18], [19].

Important progresses have been made towards understanding a structural basis of Notch signaling and regulation [7], [20]. One of the key advances is the recent determination of high-resolution X-ray structures of the negative regulatory regions (NRR) of human Notch1 and Notch2 [21], [22]. The NRR consists of three Lin12/Notch repeats (LNR) and the heterodimerization (HD) domain. The later contains both the S1 and S2 sites near the middle and the C-terminus, respectively (Fig. 1). The X-ray structure (Fig. 1b) reveals that the NRR maintains an auto-inhibited, compact fold where the LNR modules wrap around the HD domain and shield the S2 site from metalloprotease access. Clearly, large-scale conformational movements involving a displacement of LNR-A, the LNR-AB linker, and LNR-B from the HD domain are necessary for an ADAM protease to gain access to the S2 site. Several models have been proposed to explain how ligand binding might facilitate large conformational changes within the NRR, including the allosteric model and the mechanotransduction model [6], [7]. According to the allosteric model, ligand binding leads to major rearrangements of the LNR modules with respect to the HD domain. An allosteric mechanism is more likely to occur in receptors with relatively short extracellular domains, such as Notch receptors in *C.elegans*. In mammals and flies, however, where the extracellular domains of Notch receptors contain a large number (29–36) of epidermal growth factor (EGF) repeats and where the ligand binding site (EGF repeats 11–12) is positioned relatively far from the NRR, such long-range allosteric changes seem less likely.

The mechanotransduction model exploits the fact that a receptor-bound DSL ligand undergoes endocytosis into the signal-sending cell and pulls on the Notch receptor present in the signal-receiving cell. The Notch ectodomain is effectively *trans*-endocytosed into the ligand-expressing cell, while its transmembrane and intracellular domains remain associated with the receptor-expressing cell [23], [24]. Thus, the mechanotransduction model proposes that ligand endocytosis generates a mechanical strain on the NRR region and leads to “peeling off” the protective LNR modules from the HD domain [21], [22]. This model is fully consistent with previous studies demonstrating that ligand endocytosis is indeed necessary for Notch activation [25], [26] and that free ligands are generally not capable of activating Notch receptors [27], [28]. It has to be noted, however, that the requirement for ligand endocytosis has been also interpreted as a need for additional ligand processing within the endocytic compartment in order to potentiate the ligand signaling activity [29], [30], which is not related to receptor pulling.

Regardless of the mechanism involved in the rearrangement of the LNR modules during ligand-induced Notch activation, the location of the S2 site within the folded HD domain poses additional barrier for the access by ADAM metalloproteases. The S2 site is buried in a hydrophobic groove (in the middle of strand β5 beneath helix α3; see Fig. 1b). While the specific structural signatures for recognition by ADAM metalloproteases are yet to be established [31], the active sites of these proteases lie in deep clefts [32]. Clearly, unwrapping of the LNR modules alone is not sufficient to provide proteolytic access to the scissile bond at the S2 site. It has been postulated that either local or global unfolding of the HD domain must follow the displacement of the LNR modules to expose the S2 site [22]. However, the driving force and possible mechanisms for either local or global unfolding of the HD domain are not known.

In this work, we exploited topology-based coarse-grained and physics-based atomistic molecular dynamics (MD) simulations to study the conformational dynamics of the Notch NRR at equilibrium and under mechanical stress. Such computer simulations can provide a unique opportunity to directly observe protein unfolding pathways and potential short-lived intermediate states that are not accessible experimentally [33]–[35]. In particular, steered molecular dynamics (SMD) using simple topology-based coarse-grained models has been shown to be capable of reliable description of mechanically induced unfolding of proteins [36]. Topology-based modeling is built on the conceptual framework of minimally frustrated energy landscapes [37],[38]. Minimal frustration argues that natural proteins achieve efficient and robust folding by possessing largely smooth, funneled underlying free energy landscapes. There is a strong correlation between the fraction of native contacts formed and free energy. That is, native interactions largely shape the protein energy landscape. Therefore, for a given protein with known structure, one might construct simple effective energy functions by taking only native interactions into account. These topology-based models are often referred to as Gō or Gō-like models. Impressive correspondence between experiment and theory for folding mechanisms has been demonstrated for many proteins [39], [40], substantiating the notion that protein topology dictates gross aspects of the folding mechanism (e.g., order of events in folding).

We first investigated the unfolding pathway of human Notch1 NRR under mechanical force using a Gō-like model derived from the X-ray structure. SMD simulations show that pulling on the N- and C-termini of human Notch1 NRR lifts the LNR modules from the HD domain in a well defined, largely sequential fashion with the N-terminal LNR-A detached first. This is consistent with intuitive expectations based on the examination of the NRR fold alone [21], [22], as well as the proposed mechanical mechanism of Notch activation. The tertiary structures of all domains of the NRR, particularly that of the HD domain, remain largely intact throughout these initial stages of disengaging the LNR modules. Therefore, the S2 site remains inaccessible to ADAM proteases. Interestingly, the simulations predict that continuing to stretch the NRR would eventually induce local unfolding of the HD domain before dissociation of the Notch1 heterodimer. The local unfolding mainly involves the N-terminal strand β5, and the S2 site becomes fully exposed in this intermediate state. Therefore, we postulate that the putative mechanical force generated by ligand endocytosis does not only detach the protective LNR modules from the HD domain, but also further drives local unfolding of the HD domain in a functionally relevant fashion that would provide full protease access to the S2 site. We further tested this conjecture by examining the folding free energy surface of the HD domain, as well as intrinsic conformational dynamics of the HD domain with and without the LNR modules using physics-based atomistic simulations. The results support that the HD domain possesses inherent structural and dynamic characteristics that could enable it to provide additional mechanosensing necessary for Notch activation.

While this work was under review, a related study was published by Tiyanont et al. on the Notch1 NRR unfolding induced by Ca^2+^ chelation and detected by the hydrogen-exchange mass spectroscopy (HX-MS) [41]. The results indicate that the HD domain remains stable after EDTA-induced unfolding of the LNR repeats, which is also suggested by our atomistic and coarse-grained simulations. A previous NMR study on the human Notch2 HD domain alone also suggests that the whole HD domain is stably folded with and without S1 cleavage [9]. More interestingly, the HX-MS data also reveal elevated deuteration in and near the S2 region. Although significant differences in the NRR unfolding pathway may exist when unfolding is induced by a chemical agonist or by a mechanical force, the results reported by Tiyanont et al. are remarkably consistent with one of the key predictions of our simulations, that the HD domain has the inherent ability to partially unfold at the C-terminal strand.

## Results

### Characterization of the Gō-like model of human Notch1 NRR

**Figure 2:**
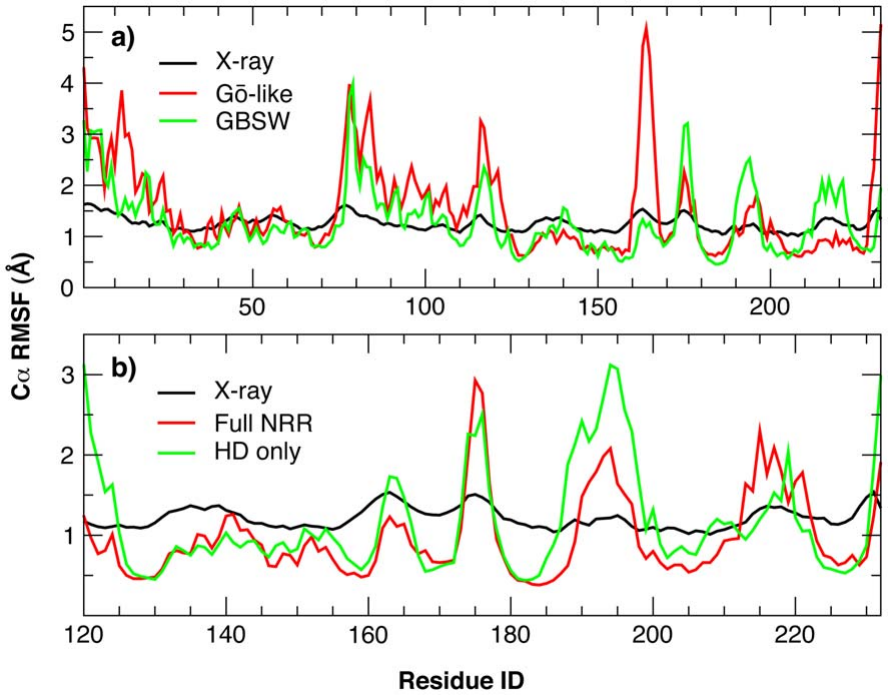
Residue RMSF profiles. a) Cα RMSF profiles calculated from the Gō-like and GBSW simulations, in comparison with the one derived from the X-ray B-factors. The final 80% of the trajectories (1.0 µs for Gō-like and 20 ns for GBSW) were used in the RMSF calculations. All snapshots were globally aligned to minimize the RMSD with respect to the native structure. The crystal B factors were converted into RMSF values using the relationship B-factor = 8π^2^(RMSF)^2^/3. b) Cα RMSF profiles for the HD domain only, calculated from the last 16 ns of the 20 ns GBSW simulations of the full NRR and HD domain only. Note that, to better reflect the fluctuations within the HD domain with and without the LNR modules, the RMSF profile for the full NRR simulation was recalculated by aligning all snapshots to minimize the RMSD of the HD domain (instead of the full NRR in Panel a). Residue ID 1–174 and 175–232 shown correspond to residues 1449–1622 and 1670–1727 in human Notch1 numbering, respectively.

As detailed in the Materials and Methods, a “sequence-flavored” coarse-grained Gō-like model [42] of human Notch1 NRR was first constructed based on the X-ray structure (PDB: 3eto) [22], and then modified to include the disulfide bonds and bound calcium ions. A 1.0 µs equilibrium simulation at 300 K was performed to verify the structural stability and to characterize the dynamic properties of the NRR. The results show that the protein is stable in the Gō-like coarse-grained effective potential as designed, with the root-mean-square deviation (RMSD) from the native structure fluctuating around 3 Å throughout the simulation (data not shown). The calculated root-mean-square fluctuation (RMSF) profile, shown in Fig. 2a, agrees qualitatively with the one derived from the crystal B-factors. This is expected, as the native structure is well conserved during the simulation. However, the coarse-grained simulation does appear to yield significantly higher fluctuation in loop regions as well as LNR-A and LNR-C regions. Importantly, higher (loop) flexibility does not appear to be an artifact of coarse-graining. An atomistic simulation using the GBSW protein force field (see Methods and Materials) yields a similar RMSF profile with comparable magnitudes of fluctuation throughout the protein, except in the loop (mapped to residue ID162–165) connecting the second and third strands of the HD domain (also see Fig. 1b). This particular loop has few native contacts with the rest of the protein, and therefore its flexibility is exaggerated in the Gō-like model. Nonetheless, it locates on the opposite side of the HD domain from where LNR modules pack. An elevated loop dynamics in this region is not expected to have significant influence on unfolding of either the NRR fold or the HD domain. Therefore, the Gō-like model as constructed is capable of realistically describing both the structure and dynamics of human Notch1 NRR.

### Force-induced unfolding of the NRR

Constant velocity SMD simulations were carried out to examine the force-induced unfolding mechanism of the NRR. The external force was applied on the N- and C-termini. This setup was designed to mimic the situation where endocytosis of the Notch-bound ligand could exert mechanical stress on the NRR. Initial simulations with different pulling speeds suggest that there is a dependence of force magnitude on the pulling speed. However, the overall unfolding pathway appears to be insensitive to pulling speeds ranging from 0.1 to 100 Å/ns, except that faster pulling tends to smear the fine details. Therefore, the final simulations were carried out with a modestly slow pulling speed of 1.0 Å/ns. This provides a good compromise between resolution and computational efficiency. Note that the simulated pulling speed is several orders of magnitude faster than that in either typical single molecule pulling experiments or the putative endocytosis-driven pulling of Notch. However, previous studies have demonstrated that this does not prevent a reliable description of mechanically induced unfolding of proteins [33], [36].

**Figure 3:**
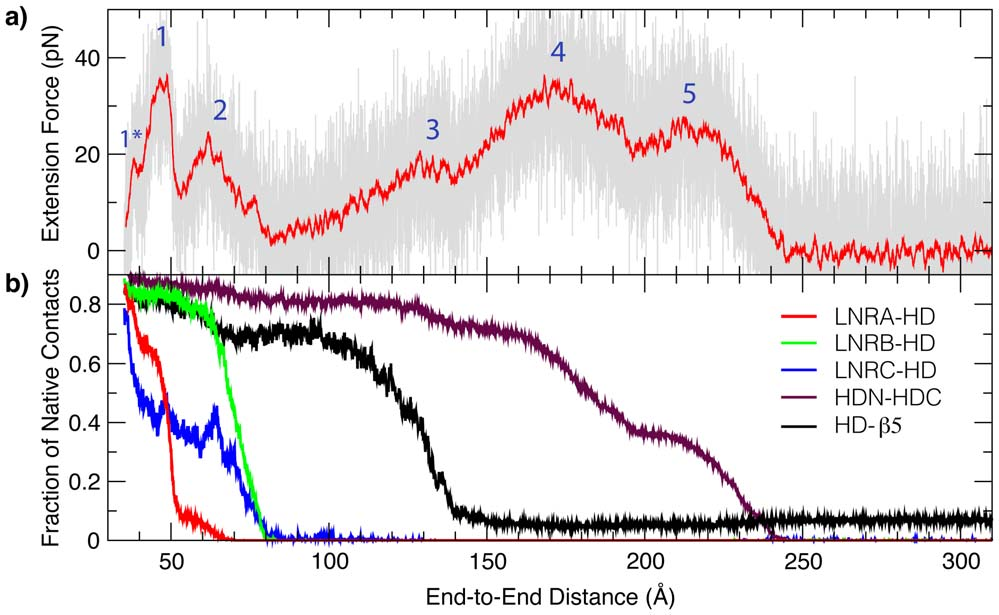
Calculated extension force profile and corresponding native fractions of the NRR. a) Average extension force as a function of the end-to-end distance computed from 60 independent coarse-grained SMD pulling simulations. The red trace is the 50-point running average. The major peaks discussed in the main text are marked. b) Average fractions of native contacts formed between different sub-domains of NRR (see Materials and Methods for domain definitions). The LNR-A module only makes a few direct contacts with the HD domain, and the native fraction shown was calculated by including all contacts with the HD domain from the first 40 residues. The HD-β5 trace was computed by considering all native contacts made by residues 1721–1727.

**Figure 4:**
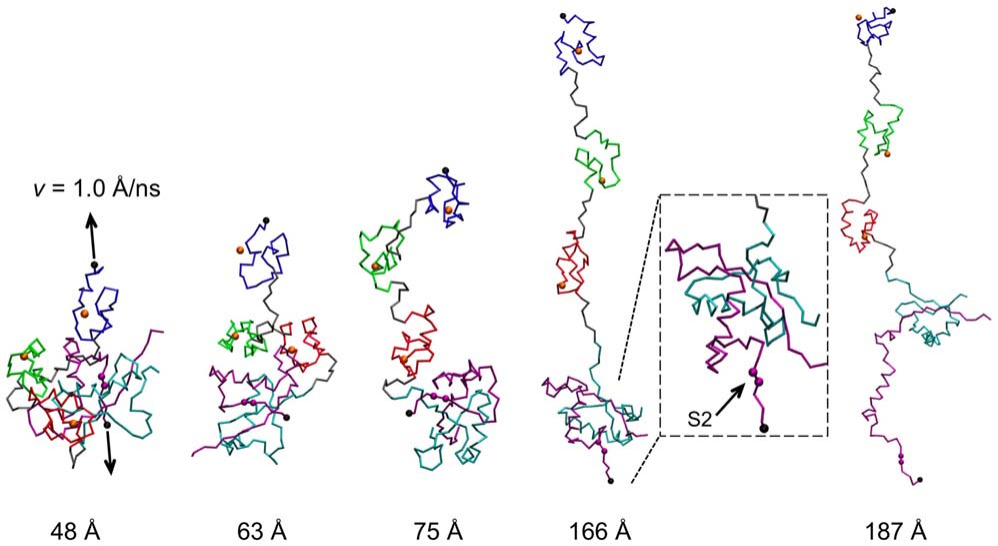
Representative snapshots of the human Notch1 NRR along the force-induced unfolding pathway during one of the SMD pulling simulations. The LNR-A, LNR-B and LNR-C modules are colored in blue, green and red, respectively, and the HDN and HDC subunits of the HD domain in cyan and purple, respectively. The N- and C-terminal Cα beads where the external force is exerted are shown in black spheres. The S2 site is marked using purple spheres. Approximate end-to-end distances for the snapshots are also listed. Note that the magnified view of the 166 Å snapshot is slightly rotated to better show the exposure of the S2 site.

Fig. 3 plots the average extension force computed from 60 independent SMD runs, together with average fractions of native contacts between various domains and subdomains of the NRR. Representative snapshots along the unfolding pathway are shown in Fig. 4. The average force profile shows several distinct major peaks, designated as Peaks 1–5 in Fig. 3a. Clearly, the NRR responds sensitively to mechanical stretching: increase of the end-to-end distance (distance between N- and C-terminal C_α_ atoms) from the native value of ∼35 Å can only be achieved via tertiary unfolding and requires substantial external force. The first major force peak, Peak 1, locates near an end-to-end displacement of 47 Å. The corresponding average fractions of native contacts between different regions of the NRR, shown in Fig. 3b, demonstrate that Peak 1 arises from unwrapping of LNR-A, which, more precisely, leads to Peak 1*, and the adjacent LNR-AB linker. This linker loop provides a hydrophobic plug that forms extensive contact with the HD domain and helps to protect the S2 site from metalloprotease cleavage (Fig. 1b). With a maximum of ∼40 pN, Peak 1 is one of the strongest peaks in the force profile. Once LNR-A (with the adjacent LNR-AB linker) is detached, smaller forces are necessary to lift the LNR-B and LNR-C modules from the HD domain (broad Peak 2). Force-induced unpacking of LNR-B and LNR-C occurs roughly at the same time. Nonetheless, LNR-B appears to be more tightly bound to the HD domain, while LNR-C has a weaker interaction and its mechanical removal is the least cooperative (manifested as a broad transition shown in the blue trace of Fig. 3b). The observation that disengagement of LNR-A, the LNR-AB linker, and, to a lesser extent, LNR-B, provides the strongest resistance to mechanical unfolding is consistent with previous cell-based reporter gene assays showing that the removal of all these three segments facilitates ligand-independent activation of human Notch1 [21].

Importantly, all LNR modules and the HD domain remain largely folded during the initial process of peeling off the LNR modules (e.g., see Fig. 4 for a representative snapshot at 75 Å end-to-end displacement). Therefore, unwrapping the LNR modules alone is not likely to provide sufficient proteolytic access to the S2 site, as expected [21], [22]. Further pulling continues to stretch the entropic loops connecting these sub-domains. Interestingly, the next key unfolding event is predicted to be local unfolding of the C-terminal strand β5, which occurs in a cooperative fashion when the extension force approaches ∼20 pN (Peak 3 in Fig. 3a). As illustrated by a representative snapshot shown in Fig. 4 (with ∼166 Å end-to-end distance), local unfolding of β5 fully exposes the S2 site for proteolytic access. This suggests that mechanical force generated by ligand endocytosis is capable of fully relieving the auto-inhibition for Notch activation. Eventual disassociation of the Notch heterodimer is predicted to occur in two stages, mainly involves unfolding of the secondary structure elements α3 and β4, respectively. The two-stage dissociation leads to two broad force peaks (Peaks 4 and 5 in Fig. 3a). It is noteworthy that heterodimer dissociation requires an extension force of ∼40 pN, considerably higher than what is required to locally unfold the β5 strand. This suggests that the intermediate state with exposed S2 site can persist for a significant period of time before global unfolding and heterodimer disassociation. An important implication is that the partially unfolded state might be required for specific recognition by an ADAM metalloprotease.

### Intrinsic folding characteristics of the HD domain

**Figure 5:**
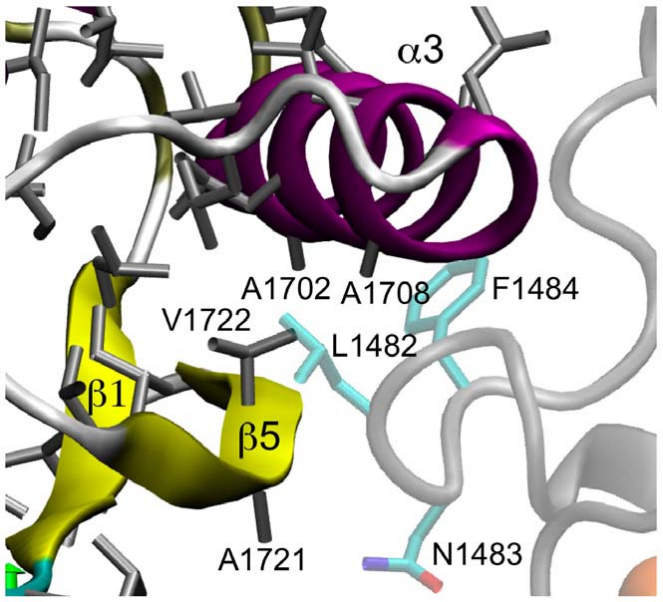
Local structural features near the HD domain strand β5. All heavy atoms of hydrophobic side chains of the HD domain are shown in gray sticks. The LNR modules are drawn in semi-transparent colors to illustrate a lack of stabilizing contacts of strand β5 in the absence of the LNR-AB liner. The key residues discussed in the main text are labeled.

The SMD pulling simulations reveal that, in addition to the auto-inhibited NRR fold, the HD domain also plays a central role in mechanosensing and Notch activation. To better understand the intrinsic folding characteristics of the HD domain, we first analyzed the structural basis of the observed mechanical weakness of the C-terminal strand β5. As illustrated in Fig. 5, strand β5 only contains two small hydrophobic residues (A1721 and V1722), while all other strands in the HD domain contain several and larger hydrophobic residues. For example, the adjacent strand β1 contains six consecutive leucine and valine residues from L1575 through L1580. Furthermore, strand β5 is loosely packed with a face of the C-terminal helix α3 that is lined with alanine and glycine residues (A1697, A1702, G1795, A1708) and thus completely lacks any large (hydrophobic) side chains that could provide stabilizing contacts with strand β5. Instead, this local void in the hydrophobic core is filled by L1482 and F1484 from the LNR-AB linker (see Fig. 5). In addition, the LNR-AB further stabilizes the NRR fold through a hydrogen bonding interaction from N1483 to E1720 on the loop that precedes the HD strand β5. These structural features fully explain substantial local mechanical weakening of strand β5 once the LNR modules, particularly, the LNR-AB linker, are disengaged from the HD domain.

**Figure 6:**
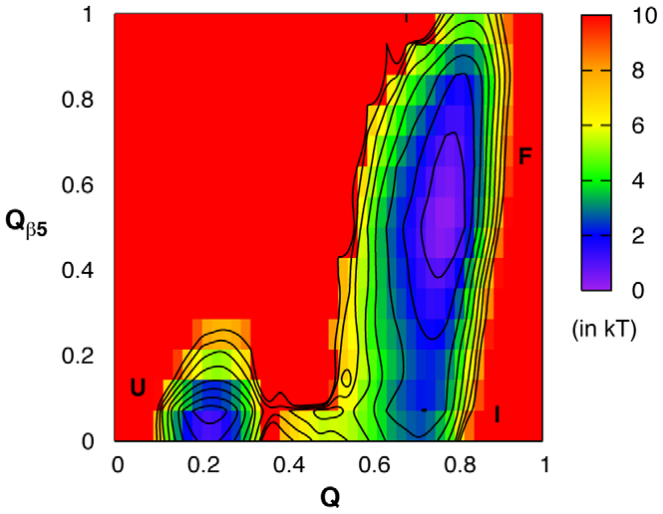
Folding free energy surface of the HD domain. The surface is shown as a function of the total native fraction (*Q*) and fraction of native contacts formed by strand β5 (*Q*_β5_) near the melting temperature. The free energy surface was calculated from a 1-ms REX-MD simulation using the Gō-like model. Contours are drawn at every kT. Approximate locations of the unfolded (U), intermediate (I), and folded (F) states are marked.

We further examined the folding free energy surfaces of the HD domain using the Gō-like model. A 1.0-µs replica exchange MD (REX-MD) simulation was preformed to extensively sample the accessible conformational space and allow converged free energy surfaces to be computed using the weighted histogram analysis method (WHAM) [43]–[45]. Fig. 6 plots the free energy surface near the melting temperature (*T*_m_∼350 K), as a function of the fraction of all native contacts (*Q*) and that of native contacts formed by strand β5 (*Q*_β5_). It reveals that the HD domain naturally unfolds through a weakly stable intermediate state (marked as I in Fig. 6), where strand β5 is largely unfolded (*Q*_β5_∼0.1) while the rest of the protein is still mostly folded. Importantly, transition between the intermediate and unfolded states involves a free energy barrier that is substantially higher than that between the folded and intermediate states. This is consistent the observation that significantly higher extension force is required to fully unfold (and disassociate) the HD domain. Furthermore, the intermediate state identified in the equilibrium calculation is similar to the one observed in force-induced unfolding trajectories (e.g., see Fig. 3 and Fig. 4). Therefore, external mechanical force induces a natural unfolding pathway of the HD domain that provides transient proteolytic access to the S2 site. In other words, the HD domain possesses inherent folding characteristics that enable it to respond to mechanical stress in a functionally relevant way.

### Intrinsic conformational dynamics of the HD domain

**Figure 7:**
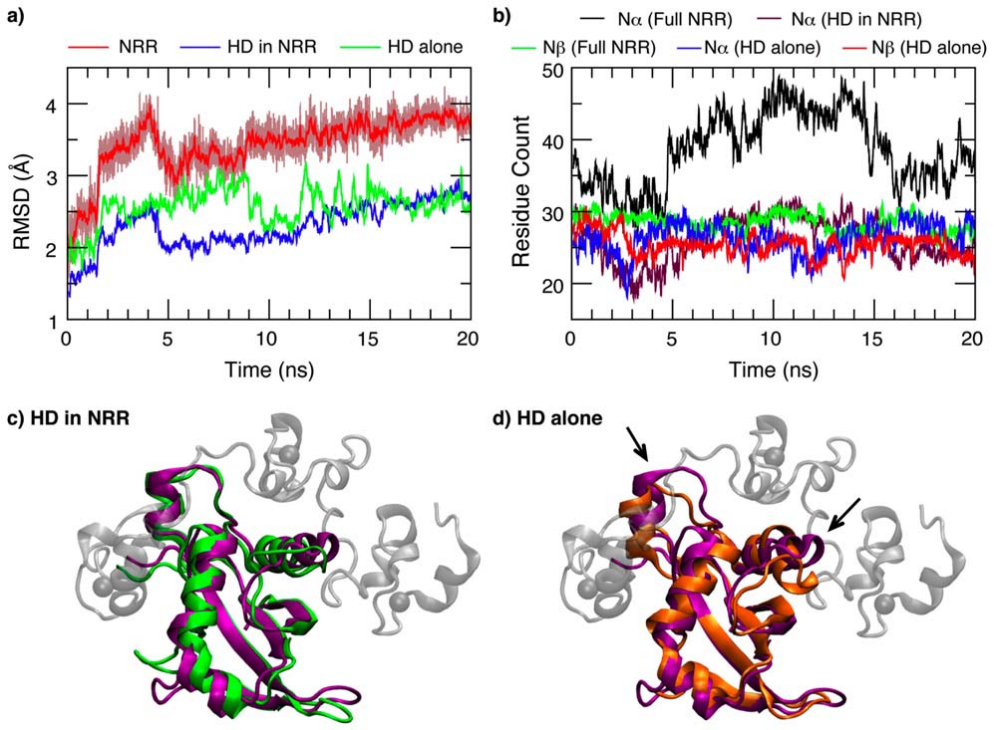
Structural properties of the HD domain with and without the LNR modules in atomistic simulations. a) The backbone RMSD as a function of the simulation time. Except for the full NRR, only 50-point running averages are plotted for clarity. b) The number of residues in α or β secondary structures as a function of time. c) The average structure of the HD domain calculated from the last 16 ns of the GBSW simulation of the full NRR (green). The X-ray structure (PDB: 3eto) is shown for comparison, where the HD domain is drawn in purple and the LNR modules in light gray. The backbone RMSD between the average and X-ray structures is 2.1 Å for the HD domain. d) The average structure of the HD domain computed from the last 16 ns of the GBSW simulation of the HD domain alone. The backbone RMSD from the X-ray structure is 2.4 Å.

Two 20 ns atomistic simulations were performed to further investigate the overall motions and fluctuations of the full NRR and the HD domain alone, respectively. The purpose is to explore potential links between dynamics and mechanosensing. An optimized GBSW implicit solvent force field [46] was used for computational efficiency. As summarized in Fig. 7, both the NRR and the HD domain alone were stable throughout the 20 ns simulation timescale. The backbone RMSD from the X-ray structure of the NRR quickly increased and stabilized around 3.5 Å. The moderate RMSD value is mainly due to fluctuations in loops and tertiary packing between the LNR repeats and the HD domain. The backbone RMSD of the HD domain itself from the X-ray structure is much smaller, ∼2.5 Å, with or without the LNR modules (green and blue traces in Fig. 7a). All secondary structure elements in the HD domain were also well maintained during both simulations (Fig. 7b). Average structures computed from the last 16 ns of the 20 ns trajectories, shown in Fig. 7c and 7d, illustrate that the HD domain itself is stable. Absence of the LNR modules only leads to small local conformational changes at the interface, with one of the most notable changes being a slight distortion of helix α3 (marked by arrows in Fig. 7d). The helix α3 distortion is apparently a direct consequence of the hydrophobic void created by removal of the LNR-AB linker (Fig. 5). While these atomic simulations with limited length are not sufficient to establish the protein stabilities, they are consistent with earlier notions that the HD domain itself appears to be stable and that (mechanically) lifting of the LNR modules is not likely to provide sufficient proteolytic access to the S2 site. Furthermore, a separate simulation shows that a chain break in the S1 loop does not appear to destabilize the isolated HD domain (see Fig. S1). The prediction of a stable isolated human Notch1 HD domain has since been confirmed by the recent HX-MS study [41] and is also consistent with previous NMR studies of the human Notoch2 HD domain [9].

**Figure 8:**
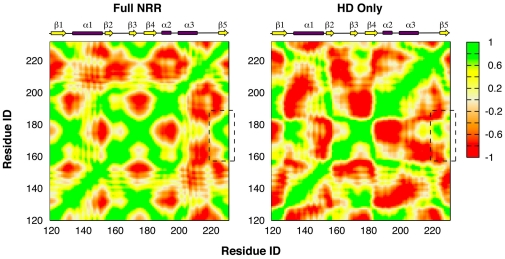
Residue-residue map of correlated motions for the HD domain with and without the LNR modules. The maps were computed as the Cα-Cα covariance matrices, extracted from the last 16 ns of the 20-ns equilibrium GBSW simulations. The dashed boxes mark regions that illustrate weaker coupling of strand β5 to the rest of the HD domain. Approximate spans of the secondary structure elements are also marked. See the Fig. 2 caption for the residue ID mapping.

Interestingly, in contrast to the apparent structural stability and insensitivity to removal of the LNR modules, internal dynamics of the HD domain appears to respond in a sensitive manner to the absence of the LNR modules. Residue RMSF profiles, shown in Fig. 2b, reveal that there is a significant increase in fluctuations in the exposed HD domain, especially at the termini and interfacial loops. Changes in protein dynamics are even more pronounced when one examines correlated motions by calculating the covariance between fluctuations of two residues. Fig. 8 compares the covariance matrices for the HD domain in the presence (Fig. 8a) and absence of the LNR modules. The covariance matrix for the full NRR is shown in Fig. S2. Clearly, there is not only a significant increase in the overall degree of dynamic coupling in the free HD domain, but the pattern of correlated motions also changes substantially. However, the C-terminal strand β5 (mapped to residue ID 226–232) becomes only weakly coupled the rest of HD domain in the absence of the LNR modules (e.g., to strands β2, β3 and β4; marked by dashed boxes in Fig. 8), while the preceding loop (mapped to residue ID 214–224) becomes more tightly coupled to adjacent structural elements and subsequently to the rest of the protein. Therefore, the HD domain responds dynamically to disengagement of the LNR modules in a fashion that it becomes poised to sense further mechanical stress via activation of an inherent unfolding pathway, namely, local unfolding of the C-terminal strand β5 to expose the S2 site prior to the disassociation of the HD domain.

## Discussion

The key event in the activation of Notch signaling is ADAM-mediated cleavage of the receptor at the S2 site. As the S2 site is buried within the HD domain and is not available for cleavage, an outstanding question remains: How does ligand engagement overcome auto-inhibition of the “off” state? Two unique features of the Notch signaling system are that the receptor and the ligand are located on two opposing cells and that receptor-ligand interaction is followed by ligand endocytosis into the ligand-expressing cell. This invites a question about force-induced conformational changes in the receptor. The mechanostransduction model of Notch activation [21] proposes that the mechanical force exerted by ligand endocytosis is instrumental in relieving the auto-inhibited conformation and exposing the S2 site for cleavage [21]. However, direct biochemical or computational data supporting this model are missing. The current study explores for the first time the effects of mechanical force on the folding and unfolding mechanism of the human Notch1 NRR using computational approaches.

Our topology-based coarse-grained modeling demonstrates that external force leads to sequential disengagement of the LNR modules from the HD domain. All these domains remain stably folded during this early stage of force induced-unfolding. Most importantly, the simulations predict that mechanical force can further drive local unfolding of strand β5 in the HD domain in a fashion that renders the S2 site exposed for cleavage. Remarkably, the partially unfolded state with exposed S2 site appears to persist for a significant period of time and, based on analysis of the underlying folding free energy surface, it represents a weakly stable intermediate inherent to the HD domain. Physics-based atomistic simulations further suggest that the β5 strand responds dynamically to the disengagement of the LNR modules and, in the absence of the LNRs, it becomes decoupled from the rest of the HD domain and thus poised to sense further mechanical stress. Taken together, these results support that the HD domain itself can provide additional mechanosensing for Notch activation and that mechanical force alone might be sufficient to fully activate Notch. However, it is also possible that the elevated spontaneous structural fluctuations alone in and near the S2 region, as suggested by HX-MS [9] and the current simulations, could provide sufficient protease access for subsequent Notch activation.

Remarkably, 40% to 50% of human T-cell acute lymphocytic leukemias harbor mutations within the NRR of Notch1 [47]–[49]. These are activating mutations, which increase ligand-independent Notch1 signaling via two different mechanisms [22], [50]. Class II mutations include insertions of ∼12 residues near the C-terminal end of the HD domain that duplicate the S2 site and directly enhance ligand-independent S2 cleavage. Class I mutations map to the HD domain and are typically single amino acid substitutions. Interestingly, while most of class I mutations lie in the hydrophobic core of the HD domain and cause its substantial destabilization, other class I mutations are positioned at the interface with the LNR domains and cause less destabilization of the HD domain [22], [50]. The activating effect of the later mutations is fully consistent with the results of our simulations, which show that the disengagement of the LNR domains changes the internal dynamics of the HD domain and facilitates the access to the S2 site.

Another interesting result of our simulations is the mechanistic and temporal resolution between the local unfolding of strand β5 and physical separation of HDN (plus LNRs) from HDC. The SMD simulations predict that the force required to disassociate the HD domain is not stronger than the force required for disengaging the LNR modules. Therefore, if endocytosis can mechanically unwrap the NRR fold, it ought to be able to disassociate the HD domain without the S2 cleavage. This is consistent with an important discovery by Nichols et al. [51] that endocytosis alone can physically disassociate the HD domain even without ADAM activity. Our simulations further indicate that, upon mechanical force, local unfolding of the β5 strand occurs first, and this step is then followed by separation of the HDN and HDC fragments. Therefore, if ADAM10/17 are available, the S2 cleavage most likely occurs prior to the dissociation of the HDN and HDC fragments. As the HDN and HDC fragments arise from the cleavage at the S1 site, our results provide further insight into the role of the S1 cleavage in Notch activation. The S1 cleavage is mediated by furin-like proteases in the Golgi and creates a discontinuity in Notch polypeptide chain transported to the cell surface. Some Notch1 mutants resistant to S1 cleavage were found to be defective of signaling when expressed in mammalian cells [51], [52]. This led to suggestion that physical separation of the extracellular and transmembrane Notch subunits during ligand endocytosis is a prerequisite for the subsequent processing at the S2 site [51]. Other reports have demonstrated, however, that while S1 cleavage mutants show impaired intracellular trafficking, they are fully capable of signaling when present at the cell surface [9]. Thus, in the context of full-length Notch1, separation of the extracellular and transmembrane domains does not seem to be required for the S2 cleavage and Notch activation. This is fully consistent with the prediction from the simulations that only local unfolding of the HD domain appears to be necessary for proteolytic access to the S2 site.

The large size of Notch receptors, their posttranslational modifications, unusually large number of extracellular domains, and a sequence of coordinated proteolytic events add to the complexity of Notch signaling and pose many challenges in both biochemical and computational analyses of Notch activation. Some of the unresolved questions are: What is the role of multiple of EGF-like repeats? Do they function to increase pulling force exerted on the NRR during ligand endocytosis? What is the effect of receptor (and ligand) glycosylation on the mechanical properties of Notch? As the extracellular domain of Notch1 has been recently shown to form dimers [53], how does the dimerization affect mechanosensing? What is the effect of oncogenic T-ALL mutations on the mechanical properties and internal dynamics of Notch1? Application of computational approaches should help answering these questions and thus complement the ongoing biochemical and cell biological characterization of Notch proteins.

## Materials and Methods

### X-ray structures and domain definitions

We chose to use the high-resolution X-ray structure of human Notch1 NRR (PDB: 3eto [22]) as the model of the auto-inhibited conformation. This structure was determined using a continuous construct without the S1 loop (residues 1623–1669) excised. The structure for the furin-cleaved Notch1 NRR is also available (PDB: 3i08 [9]), but with much lower resolution (3.2 Å vs. 2.0 Å for 3eto). Interestingly, the S1 loop excised in 3eto is also missing in 3io8, presumably due to the lack of a stable conformation. The structured regions of the two structures show little change except for a few residues at the S1 chain break site. Therefore, 3eto instead of 3i08 was used in the current study. The domain boundaries are: LNR-A: residues 1449–1480; LNR-B: residues 1491–1522; LNR-C: residues 1529–1562; and, HD: residues 1568–1727. The actual S1 cleavage would break the covalent linkage between residues 1622 and 1670 in the 3eto construct, and leads to two non-covalently associated subunits of the HD domain. These two subunits are designated as HDN (residues 1568–1622) and HDC (residues 1670–1727). The S2 site locates between A1721 and V1722, which is part of strand β5 of the HD domain.

### Topology-based coarse-grained modeling of human Notch1 NRR

A “sequence-flavored” Gō-like model [42] of human Notch1 NRR was first generated based on the X-ray structure (PDB: 3eto), using the Multiscale Modeling Tools for Structural Biology (MMTSB) Gō-Model Builder (http://www.mmtsb.org). Each amino acid residue is represented as a single coarse-grain C_α_ bead connected via virtual bonds (Fig. 1c). The mass of each bead is taken to be that of the corresponding amino acid residue. As in a typical Gō-model, all virtual bonds and angles are derived directly from the native structure, and non-bonded C_α_ bead interact favorably only between pairs of residues that are in contact in the native structure. The native contacts include backbone hydrogen bonding as well as side chain interactions. However, unlike conventional Gō-models where all native interactions contribute uniformly to the total energy, the sequence-flavored” Gō-like model incorporates the Miyazawa-Jernigan (MJ) statistical potentials [54] to provide residue-specific energetic biases for native interactions. In addition, the model includes knowledge-based, sequence-dependent, but native-structure independent, pseudo-torsional potentials. Such sequence flavoring has been shown to enhance the realism of simple Gō-like models and provides the ability to recapitulate subtle differences in folding mechanisms of topologically analogous proteins [42], [55]–[57].

The original model generated by the MMTSB Gō-Model Builder was then modified to include the bound calcium ions and disulfide bonds. Human Notch1 NRR contains a total of ten disulfide bonds (see Fig. 1), three in each LNR module and one in the HD domain. These disulfide bonds are modeled using harmonic distance restraint potentials with a force constant of 1.0 kcal/mol/Å^2^. These harmonic restraints are sufficient to maintain the bond linkages, while providing sufficient flexibility for conformational sampling. The bound calcium ions are coordinated by six to seven carbonyl groups from the peptide backbone and aspartic acid side chains. Here, each calcium ion is represented as a neutral atom with a radius of 2 Å. Coordination with the carbonyl groups is treated as additional native interactions. The strength of calcium ions with carbonyl groups was chosen to be 2.0 kcal/mol. This parameter was chosen empirically by examining the stability of calcium ion coordination during equilibrium simulations. In addition, to prevent occasional dissociation and loss of calcium ions (such as during high-temperature and/or SMD pulling simulations), weak harmonic distance restraints were imposed between the ions and one of the coordinating carbonyl groups to contain the ions within the proximity of their native binding sites. Since there is no evidence that tertiary unfolding of the LNR modules is implicated in Notch activation, the precise approach of maintaining disulfide bonding and calcium coordination is not expected to affect the force-induced unfolding simulations. By construction, the protein has a melting temperature of about 350 K and is stably folded at 300 K. Finally, to directly observe the Notch heterodimer disassociation, the bond between residues 1622 and 1670 was deleted during SMD pulling simulations. All the other simulations were carried out with a single continuous peptide chain as used in the X-ray structure determination unless otherwise noted.

### Equilibrium, SMD and REX-MD simulations using the Gō-like model

Equilibrium and SMD simulations were performed using CHARMM [58], [59]. Langevin dynamics was performed with a time step of 10 fs and a friction coefficient of 0.1 ps^−1^. Lengths of all virtual bonds were kept fixed using SHAKE [60], and the cutoff for non-bonded interactions was 25 Å. Constant velocity SMD was performed using the AFM module [61] in CHARMM. Multiple pulling speeds ranging from 0.1 to 100 Å/ns were tested before a final speed of 1.0 Å/ns was chosen to provide a compromise between efficiency and resolution. A total of 60 independent SMD runs of 500 ns each were carried out at 300 K (see Movie S1 for a representative pulling simulation).

REX-MD was used to enhance conformational sampling and allow converged folding free energy surfaces to be calculated. REX is an advanced sampling technique that has proven extremely powerful in protein simulations [62]. The basic idea is to simulate multiple independent replicas of the system at different temperatures. Periodically, replicas attempt to exchange simulation temperatures according to a Metropolis criterion that preserves the detailed balance and ensures proper canonical ensembles at all temperatures. The resulting random walk in the temperature space helps the system to avoid being kinetically trapped in states of local energy minima. The REX-MD simulation was performed using the MMTSB toolset [63] and CHARMM. Eight replicas were distributed exponentially from 300 to 400 K. Exchange of simulation temperatures between neighboring replicas was attempted after every 10 ps, and the total simulation length was 1.0 µs (100,000 REX cycles).

### Atomistic implicit solvent simulations

All atomistic simulations were carried using a previously optimized GBSW implicit solvent protein force field [46], [64] based on the CHARMM22/CMAP all-atom force field [65]–[68]. Implicit solvent is essentially a coarse-grained approach where only the protein is represented at atomic level, and the mean influence of solvent is captured by the free energy cost of solvating the protein (thus solvent is implicit) [69]. The system size is reduced about 10 fold compared to traditional explicit solvent, and thus further facilitate facile sampling of accessible conformation space [70]. Importantly, this particular GBSW force field has been extensively optimized to reproduce the experimental structures and stabilities of a range of helical peptides, β-hairpins, and mini-proteins [46]. The same force field has also been successfully applied to describe the conformational equilibria of several proteins under stable and unstable conditions [71]–[75]. Therefore, it allows computationally efficient yet reliable simulations of human Notch1 NRR. Both the full NRR and HD domain were simulated at 300 K for 20 ns after proper energy minimization and short MD equilibration. The termini were blocked with acetyl (Ace) and amine (NH_2_) groups. Default GBSW parameters were used [46]. SHAKE [60] was applied to fix lengths of all hydrogen-involving bonds to allow a dynamics time step of 2 fs. Both protein constructs stabilize rapidly in a few ns, and all structural and dynamic properties examined in this work appear to converge well. Final analysis was done using the last 16 ns trajectories.

### Data Analysis

All the analysis was carried out using CHARMM, the MMTSB toolset, and additional in-house scripts. For coarse-grained modeling, a given native contact is considered formed if the inter-C_α_ distance is no more than 1 Å greater than the native distance. Folding free energy surfaces were calculated by combining informational from all temperatures from the REX-MD simulation using WHAM [43]–[45]. All structural visualizations presented in this work were prepared with the VMD program [76].

## Supporting Information

Figure S1 **Structural properties of the isolated HD domain with and without a chain break in the S1 loop during 20-ns atomistic simulations.** a) The backbone RMSD as a function of the simulation time. b) The number of residues in α or β secondary structures as a function of time. c) The final snapshot and d) the average structure from the last 16 ns simulation of the HD domain with a break (cyan), in comparison with the X-ray structure (PDB: 3eto) (purple). The backbone RMSD from the X-ray structure is 2.2 Å for the average structure and 3.6 Å for the final snapshot. The location of the break in the S1 loop is marked. These results suggest that the break does not de-stabilize the isolated HD domain within the simulation time frame.
(TIF)

Figure S2 **Residue-residue map of correlated motions for the human Notch1 NRR.** The maps were computed as the Cα-Cα covariance matrices, extracted from the last 16 ns of a 20-ns equilibrium GBSW simulation. It shows that all LNR modules are tightly coupled with the HD domain, particularly with regions that are direct contacts. This highlights the strength of the inter-domain interactions between the LNR modules and the HD domain. Interestingly, the LNR-B module (residue ID 43–74) appears to dynamically uncoupled from both LNR-A (residue ID 1–32) and LNR-C (residue ID 81–114), possibly due to long linkers between three LNR modules.
(TIF)

Movie S1 **A movie of a representative force unfolding simulation.** The Notch1 NRR is represented using the same scheme as in Fig. 4 of the main text. Specifically, the LNR-A, LNR-B and LNR-C modules are colored in blue, green and red, respectively, and the HDN and HDC subunits of the HD domain in cyan and purple, respectively. The N- and C-terminal Cα beads where the external force is exerted are shown in black spheres. The S2 site is marked using purple spheres. N. B. while the protein is allow to freely diffuse and rotate during the pulling simulations, all snapshots are re-oriented using the HD domain in the movie to allow better visualization of the unfolding pathway. The snapshots were taken every 0.1 ns, and the movie was truncated shortly after the HD domain dissociates.
(MPG)

## References

[1] Artavanis-Tsakonas S, Muskavitch MA (2010). Notch: the past, the present, and the future.. Curr Top Dev Biol.

[2] Kopan R, Ilagan MX (2009). The canonical Notch signaling pathway: unfolding the activation mechanism.. Cell.

[3] Fortini ME (2009). Notch signaling: the core pathway and its posttranslational regulation.. Dev Cell.

[4] Aster JC, Pear WS, Blacklow SC (2008). Notch signaling in leukemia.. Annual Review of Pathology-Mechanisms of Disease.

[5] Koch U, Radtke F (2010). Notch signaling in solid tumors.. Curr Top Dev Biol.

[6] Kovall RA, Blacklow SC (2010). Mechanistic insights into Notch receptor signaling from structural and biochemical studies.. Curr Top Dev Biol.

[7] Gordon WR, Arnett KL, Blacklow SC (2008). The molecular logic of Notch signaling - a structural and biochemical perspective.. J Cell Sci.

[8] Logeat F, Bessia C, Brou C, LeBail O, Jarriault S (1998). The Notch1 receptor is cleaved constitutively by a furin-like convertase.. Proc Natl Acad Sci U S A.

[9] Gordon WR, Vardar-Ulu D, L'Heureux S, Ashworth T, Malecki MJ (2009). Effects of S1 Cleavage on the Structure, Surface Export, and Signaling Activity of Human Notch1 and Notch2.. PLoS ONE.

[10] Brou C, Logeat F, Gupta N, Bessia C, LeBail O (2000). A novel proteolytic cleavage involved in Notch signaling: the role of the disintegrin-metalloprotease TACE.. Mol Cell.

[11] Mumm JS, Schroeter EH, Saxena MT, Griesemer A, Tian X (2000). A ligand-induced extracellular cleavage regulates gamma-secretase-like proteolytic activation of Notch1.. Mol Cell.

[12] Hartmann D, de Strooper B, Serneels L, Craessaerts K, Herreman A (2002). The disintegrin/metalloprotease ADAM 10 is essential for Notch signalling but not for alpha-secretase activity in fibroblasts.. Hum Mol Genet.

[13] van Tetering G, van Diest P, Verlaan I, van der Wall E, Kopan R (2009). Metalloprotease ADAM10 is required for Notch1 site 2 cleavage.. J Biol Chem.

[14] Bozkulak EC, Weinmaster G (2009). Selective use of ADAM10 and ADAM17 in activation of Notch1 signaling.. Mol Cell Biol.

[15] De Strooper B, Annaert W, Cupers P, Saftig P, Craessaerts K (1999). A presenilin-1-dependent gamma-secretase-like protease mediates release of Notch intracellular domain.. Nature.

[16] Ye Y, Lukinova N, Fortini ME (1999). Neurogenic phenotypes and altered Notch processing in Drosophila Presenilin mutants.. Nature.

[17] Struhl G, Greenwald I (1999). Presenilin is required for activity and nuclear access of Notch in Drosophila.. Nature.

[18] Arnett KL, Hass M, McArthur DG, Ilagan MX, Aster JC (2010). Structural and mechanistic insights into cooperative assembly of dimeric Notch transcription complexes.. Nat Struct Mol Biol.

[19] Kovall RA (2007). Structures of CSL, Notch and Mastermind proteins: piecing together an active transcription complex.. Curr Opin Struct Biol.

[20] Kovall RA (2008). More complicated than it looks: assembly of Notch pathway transcription complexes.. Oncogene.

[21] Gordon WR, Vardar-Ulu D, Histen G, Sanchez-Irizarry C, Aster JC (2007). Structural basis for autoinhibition of Notch.. Nat Struct Mol Biol.

[22] Gordon WR, Roy M, Vardar-Ulu D, Garfinkel M, Mansour MR (2009). Structure of the Notch1-negative regulatory region: implications for normal activation and pathogenic signaling in T-ALL.. Blood.

[23] Chitnis A (2006). Why is delta endocytosis required for effective activation of notch?. Dev Dyn.

[24] Le Borgne R, Bardin A, Schweisguth F (2005). The roles of receptor and ligand endocytosis in regulating Notch signaling.. Development.

[25] Seugnet L, Simpson P, Haenlin M (1997). Requirement for dynamin during Notch signaling in Drosophila neurogenesis.. Dev Biol.

[26] Parks AL, Klueg KM, Stout JR, Muskavitch MA (2000). Ligand endocytosis drives receptor dissociation and activation in the Notch pathway.. Development.

[27] Sun X, Artavanis-Tsakonas S (1997). Secreted forms of DELTA and SERRATE define antagonists of Notch signaling in Drosophila.. Development.

[28] Varnum-Finney B, Wu L, Yu M, Brashem-Stein C, Staats S (2000). Immobilization of Notch ligand, Delta-1, is required for induction of notch signaling.. J Cell Sci.

[29] Wang W, Struhl G (2004). Drosophila Epsin mediates a select endocytic pathway that DSL ligands must enter to activate Notch.. Development.

[30] Wang W, Struhl G (2005). Distinct roles for Mind bomb, Neuralized and Epsin in mediating DSL endocytosis and signaling in Drosophila.. Development.

[31] Edwards DR, Handsley MM, Pennington CJ (2008). The ADAM metalloproteinases.. Mol Aspects Med.

[32] Maskos K, Fernandez-Catalan C, Huber R, Bourenkov GP, Bartunik H (1998). Crystal structure of the catalytic domain of human tumor necrosis factor-alpha-converting enzyme.. Proc Natl Acad Sci U S A.

[33] Grubmuller H, Heymann B, Tavan P (1996). Ligand binding: Molecular mechanics calculation of the streptavidin biotin rupture force.. Science.

[34] Puchner EM, Alexandrovich A, Kho AL, Hensen U, Schafer LV (2008). Mechanoenzymatics of titin kinase.. Proc Natl Acad Sci U S A.

[35] Brockwell DJ, Paci E, Zinober RC, Beddard GS, Olmsted PD (2003). Pulling geometry defines the mechanical resistance of a beta-sheet protein.. Nat Struct Biol.

[36] West DK, Brockwell DJ, Olmsted PD, Radford SE, Paci E (2006). Mechanical resistance of proteins explained using simple molecular models.. Biophys J.

[37] Onuchic JN, LutheySchulten Z, Wolynes PG (1997). Theory of protein folding: The energy landscape perspective.. Annu Rev Phys Chem.

[38] Mirny L, Shakhnovich E (2001). Protein folding theory: From lattice to all-atom models.. Annu Rev Bioph Biom.

[39] Wolynes PG (2005). Recent successes of the energy landscape theory of protein folding and function.. Q Rev Biophys.

[40] Hills RD, Brooks CL (2009). Insights from Coarse-Grained Go Models for Protein Folding and Dynamics.. Int J Mol Sci.

[41] Tiyanont K, Wales TE, Aste-Amezaga M, Aster JC, Engen JR (2011). Evidence for Increased Exposure of the Notch1 Metalloprotease Cleavage Site upon Conversion to an Activated Conformation.. Structure.

[42] Karanicolas J, Brooks CL (2002). The origins of asymmetry in the folding transition states of protein L and protein G.. Protein Sci.

[43] Kumar S, Bouzida D, Swendsen RH, Kollman PA, Rosenberg JM (1992). The Weighted Histogram Analysis Method for Free-Energy Calculations on Biomolecules .1. the Method.. J Comput Chem.

[44] Gallicchio E, Andrec M, Felts AK, Levy RM (2005). Temperature weighted histogram analysis method, replica exchange, and transition paths.. J Phys Chem B.

[45] Roux B (1995). The calculation of the potential of mean force using computer simulations.. Comput Phys Comm.

[46] Chen JH, Im WP, Brooks CL (2006). Balancing solvation and intramolecular interactions: Toward a consistent generalized born force field.. J Am Chem Soc.

[47] Weng AP, Ferrando AA, Lee W, Morris JPt, Silverman LB (2004). Activating mutations of NOTCH1 in human T cell acute lymphoblastic leukemia.. Science.

[48] Mansour MR, Linch DC, Foroni L, Goldstone AH, Gale RE (2006). High incidence of Notch-1 mutations in adult patients with T-cell acute lymphoblastic leukemia.. Leukemia.

[49] Zhu YM, Zhao WL, Fu JF, Shi JY, Pan Q (2006). NOTCH1 mutations in T-cell acute lymphoblastic leukemia: prognostic significance and implication in multifactorial leukemogenesis.. Clin Cancer Res.

[50] Malecki MJ, Sanchez-Irizarry C, Mitchell JL, Histen G, Xu ML (2006). Leukemia-associated mutations within the NOTCH1 heterodimerization domain fall into at least two distinct mechanistic classes.. Mol Cell Biol.

[51] Nichols JT, Miyamoto A, Olsen SL, D'Souza B, Yao C (2007). DSL ligand endocytosis physically dissociates Notch1 heterodimers before activating proteolysis can occur.. J Cell Biol.

[52] Bush G, diSibio G, Miyamoto A, Denault JB, Leduc R (2001). Ligand-induced signaling in the absence of furin processing of Notch1.. Dev Biol.

[53] Kelly DF, Lake RJ, Middelkoop TC, Fan HY, Artavanis-Tsakonas S (2010). Molecular structure and dimeric organization of the Notch extracellular domain as revealed by electron microscopy.. PLoS ONE.

[54] Miyazawa S, Jernigan RL (1996). Residue-residue potentials with a favorable contact pair term and an unfavorable high packing density term, for simulation and threading.. J Mol Biol.

[55] Karanicolas J, Brooks CL (2003). The structural basis for biphasic kinetics in the folding of the WW domain from a formin-binding protein: Lessons for protein design?. Proc Natl Acad Sci U S A.

[56] Karanicolas J, Brooks CL (2004). Integrating folding kinetics and protein function: Biphasic kinetics and dual binding specificity in a WW domain.. Proc Natl Acad Sci U S A.

[57] Hills RD, Brooks CL (2008). Subdomain competition, cooperativity, and topological frustration in the folding of CheY.. J Mol Biol.

[58] Brooks BR, Bruccoleri RE, Olafson BD, States DJ, Swaminathan S (1983). Charmm - a Program for Macromolecular Energy, Minimization, and Dynamics Calculations.. J Comput Chem.

[59] Brooks BR, Brooks CL, Mackerell AD, Nilsson L, Petrella RJ (2009). CHARMM: The Biomolecular Simulation Program.. J Comput Chem.

[60] Ryckaert JP, Ciccotti G, Berendsen HJC (1977). Numerical-Integration of Cartesian Equations of Motion of a System with Constraints - Molecular-Dynamics of N-Alkanes.. J Comput Phys.

[61] Paci E, Karplus M (2000). Unfolding proteins by external forces and temperature: The importance of topology and energetics.. Proc Natl Acad Sci U S A.

[62] Sugita Y, Okamoto Y (1999). Replica-exchange molecular dynamics method for protein folding.. Chem Phys Lett.

[63] Feig M, Karanicolas J, Brooks CL (2004). MMTSB Tool Set: enhanced sampling and multiscale modeling methods for applications in structural biology.. J Mol Graph Model.

[64] Im WP, Lee MS, Brooks CL (2003). Generalized born model with a simple smoothing function.. J Comput Chem.

[65] Feig M, MacKerell AD, Brooks CL (2003). Force field influence on the observation of pi-helical protein structures in molecular dynamics simulations.. J Phys Chem B.

[66] MacKerell AD, Bashford D, Bellott M, Dunbrack RL, Evanseck JD (1998). All-atom empirical potential for molecular modeling and dynamics studies of proteins.. J Phys Chem B.

[67] Mackerell AD, Feig M, Brooks CL (2004). Extending the treatment of backbone energetics in protein force fields: Limitations of gas-phase quantum mechanics in reproducing protein conformational distributions in molecular dynamics simulations.. J Comput Chem.

[68] MacKerell AD, Feig M, Brooks CL (2004). Improved treatment of the protein backbone in empirical force fields.. J Am Chem Soc.

[69] Roux B, Simonson T (1999). Implicit solvent models.. Biophysical Chemistry.

[70] Chen JH, Brooks CL, Khandogin J (2008). Recent advances in implicit solvent based methods for biomolecular simulations.. Curr Opin Struc Biol.

[71] Khandogin J, Chen JH, Brooks CL (2006). Exploring atomistic details of pH-dependent peptide folding.. Proc Natl Acad Sci U S A.

[72] Khandogin J, Brooks CL (2007). Linking folding with aggregation in Alzheimer's beta-amyloid peptides.. Proc Natl Acad Sci U S A.

[73] Khandogin J, Raleigh DP, Brooks CL (2007). Folding intermediate in the villin headpiece domain arises from disruption of a N-terminal hydrogen-bonded network.. J Am Chem Soc.

[74] Chen JH (2009). Intrinsically disordered p53 extreme C-terminus binds to S100B(betabeta) through “fly-casting”.. J Am Chem Soc.

[75] Ganguly D, Chen J (2009). Atomistic details of the disordered states of KID and pKID. implications in coupled binding and folding.. J Am Chem Soc.

[76] Humphrey W, Dalke A, Schulten K (1996). VMD: Visual molecular dynamics.. J Mol Graph.

