# Supplementary figures and images for: Force-Induced Unfolding Simulations of the Human Notch1 Negative Regulatory Region: Possible Roles of the Heterodimerization Domain in Mechanosensing
Source: PLoS One. 2011 Jul 28;6(7):e22837. doi: 10.1371/journal.pone.0022837 (PMC3145759; doi:10.1371/journal.pone.0022837)

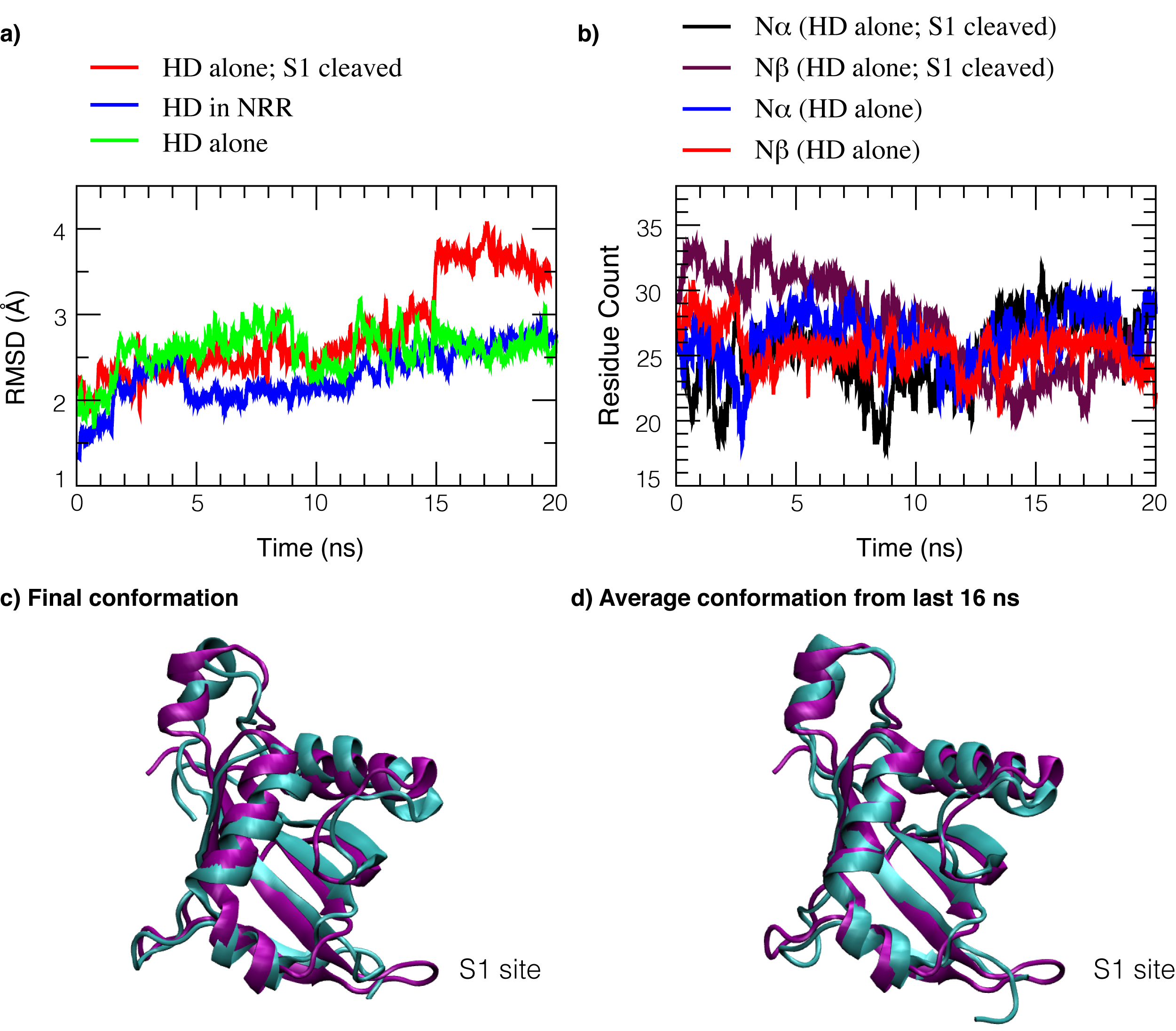

Supplement: Figure S1 — Structural properties of the isolated HD domain with and without a chain break in the S1 loop during 20-ns atomistic simulations. a) The backbone RMSD as a function of the simulation time. b) The number of residues in α or β secondary structures as a function of time. c) The final snapshot and d) the average structure from the last 16 ns simulation of the HD domain with a break (cyan), in comparison with the X-ray structure (PDB: 3eto) (purple). The backbone RMSD from the X-ray structure is 2.2 Å for the average structure and 3.6 Å for the final snapshot. The location of the break in the S1 loop is marked. These results suggest that the break does not de-stabilize the isolated HD domain within the simulation time frame. (TIF) [file pone.0022837.s001.tif]

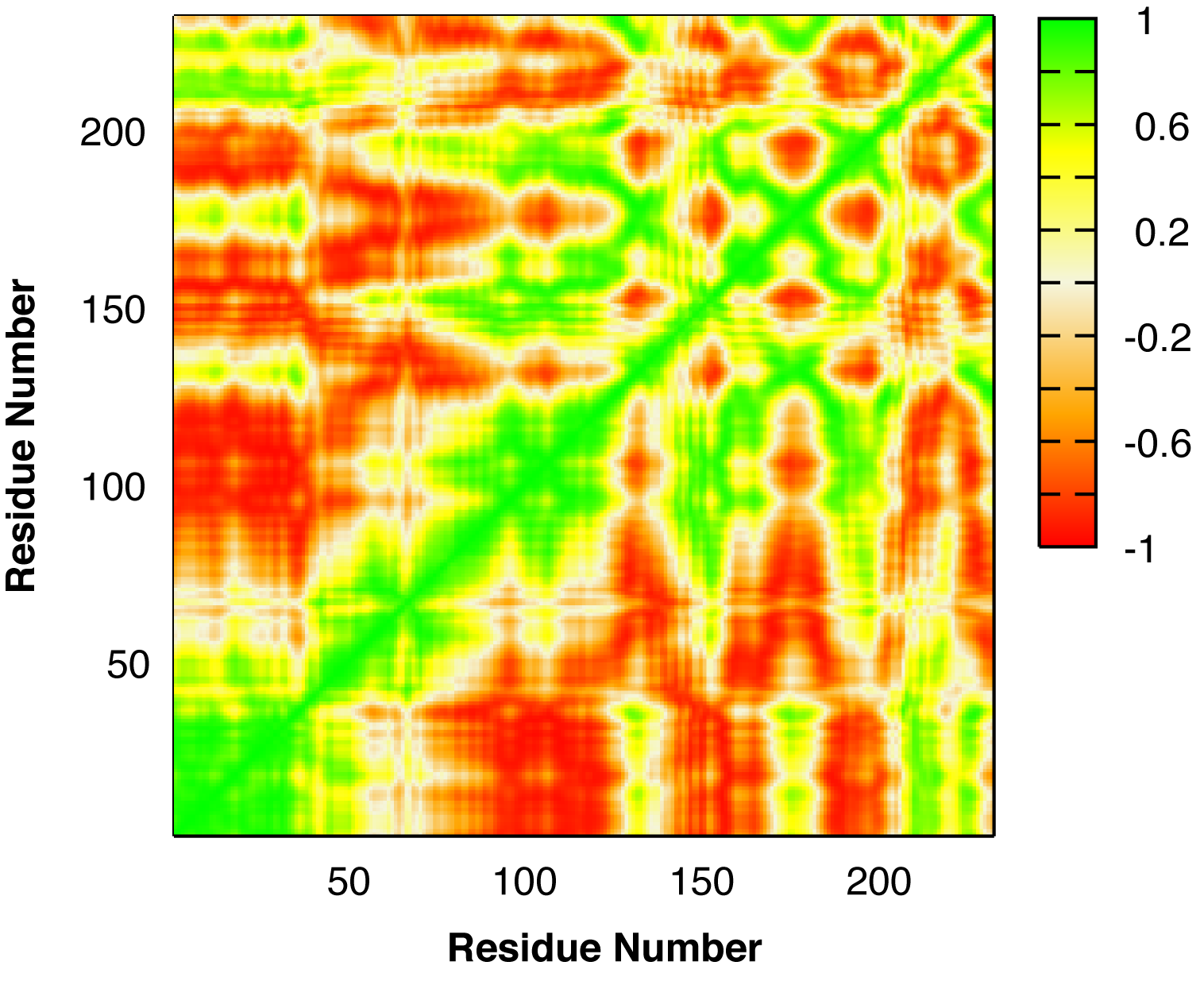

Supplement: Figure S2 — Residue-residue map of correlated motions for the human Notch1 NRR. The maps were computed as the Cα-Cα covariance matrices, extracted from the last 16 ns of a 20-ns equilibrium GBSW simulation. It shows that all LNR modules are tightly coupled with the HD domain, particularly with regions that are direct contacts. This highlights the strength of the inter-domain interactions between the LNR modules and the HD domain. Interestingly, the LNR-B module (residue ID 43–74) appears to dynamically uncoupled from both LNR-A (residue ID 1–32) and LNR-C (residue ID 81–114), possibly due to long linkers between three LNR modules. (TIF) [file pone.0022837.s002.tif]
